# Improving Mechanical and Barrier Properties of Antibacterial Poly(Phenylene Sulfide) Nanocomposites Reinforced with Nano Zinc Oxide-Decorated Graphene

**DOI:** 10.3390/polym15132779

**Published:** 2023-06-22

**Authors:** Chi-Hui Tsou, Jian-Hua Du, Wei-Hua Yao, Lei Fu, Chin-San Wu, Yuxia Huang, Chang-Lei Qu, Bin Liao

**Affiliations:** 1 School of Materials Science and Engineering, Sichuan University of Science and Engineering, Zigong 643000, China; 2 Material Corrosion and Protection Key Laboratory of Sichuan Province, Sichuan University of Science and Engineering, Zigong 643000, China; 3 Sichuan Bozhiduo Technology Co., Ltd., Chengdu 610599, China; 4 Sichuan Zhixiangyi Technology Co., Ltd., Chengdu 610599, China; 5 Department of Materials and textiles, Asia Eastern University of Science and Technology, New Taipei City 220, Taiwan; 6 School of Mechanical Engineering, Sichuan University of Science and Engineering, Yibin 644005, China; 7 Department of Applied Cosmetology, Kao Yuan University, Kaohsiung 82101, Taiwan

**Keywords:** polyphenylene sulfide (PPS), nano zinc oxide-decorated graphene (G-ZnO), mechanical properties, antibacterial nanocomposites, barrier performance

## Abstract

Nano zinc oxide-decorated graphene (G-ZnO) was blended with polyphenylene sulfide (PPS) to improve its tensile, thermal, crystalline, and barrier properties. The properties of neat PPS and PPS/G-ZnO nanocomposites were characterized and compared using various tests, including tensile tests, scanning electron microscopy, X-ray diffraction, differential scanning calorimetry, thermogravimetric analysis, evaluation of Escherichia coli inhibition, and barrier performance. The results demonstrated that G-ZnO played a crucial role in heterogeneous nucleation and reinforcement. When the concentration of G-ZnO was 0.3%, the tensile strength, elongation at break, thermostability, crystallinity, and water vapor permeability coefficients (WVPC) approached their maximum values, and the microscopic morphology changed from the original brittle fracture to a relatively tough fracture. In addition, when G-ZnO was added to PPS at a ratio of 0.3%, the tensile strength, elongation at break, and WVPC of PPS were increased by 129%, 150%, and 283%, respectively, compared to pure PPS. G-ZnO endowed the nanocomposites with antibacterial properties. The improvement in barrier performance can be attributed to three reasons: (1) the presence of G-ZnO extended the penetration path of molecules; (2) the coordination and hydrogen bonds between PPS polymer matrix and G-ZnO nanofiller narrowed the H_2_O transmission path; and (3) due to its more hydrophobic surface, water molecules were less likely to enter the interior of PPS/G-ZnO nanocomposites. This study provides valuable insights for developing high-performance PPS-based nanocomposites for various applications.

## 1. Introduction

Polyphenylene sulfide (PPS) is a polymer with a repeating structure unit of phenylene sulfide in the molecule, and it is a new type of functional engineering plastic with high performance [1]. PPS reinforced with fillers is widely used in engineering composites because it can significantly enhance its properties. Carbon materials, such as carbon fibers (CF) [2], nanodiamond [3], carbon nanotubes [4], fullerenes [5], graphite [6], carbon black [7], and graphene [8], are commonly used fillers in polymer composites. After the incorporation of carbon materials into PPS polymer, the performance of composites can be enhanced.

The mechanical properties of composite materials are influenced by interfacial adhesion between the carbon material and the polymer. When there is strong adhesion at the interface, the mechanical properties of the composite material are generally improved. This is because interfacial adhesion affects the transfer of stress between the carbon material and the polymer [9]. Uematsu et al. suggested that the high interfacial adhesion observed between PPS and CF is due to the alignment of PPS molecular chains at the interphase, which is facilitated by the π-π interaction between PPS and CF [10]. Dydek et al. investigated the impact of processing temperature and carbon nanotube (CNT) content on the properties of polyphenylene sulfide (PPS)-based nanocomposites. The findings indicated that incorporating CNT into PPS resulted in an increase in both crystallinity and crystallization temperature. The highest thermal diffusivity and conductivity values were observed in nanocomposites containing 8 to 10 wt% CNT. Meanwhile, when the CNT content was 2%, the resulting nanocomposites exhibited good elasticity [11]. Yu et al. prepared PPS-based composites by incorporating CNTs as fillers. Their results indicated a significant improvement in the thermal and mechanical properties of the composites with increasing CNT content [12].

Graphene is a type of 2D material [13] that possesses high crystal and electronic quality. Due to its unique optical, physical, electrical, and chemical properties, a variety of semiconductor composite photocatalysts supported by 2D graphene nanosheets or filled with graphene have been widely developed and applied in various fields, as demonstrated in several studies [14,15,16]. In particular, graphene’s excellent electrical and thermal conductivity and high strength make it a suitable reinforcement for polymers, as it can significantly enhance their mechanical and thermal properties [17,18,19].

Despite its advantageous properties, graphene has poor compatibility with polymer matrices due to its lack of functional groups. To address this issue, Zhao et al. developed a method to prepare graphene-like nanomaterials from expanded graphite through ultrasonic treatment, which were then used to fill PPS and create nanocomposites [20]. However, the results indicated that the addition of graphene-like nanomaterials increased the crystallization rate of PPS but reduced its bending strength, likely due to poor interfacial compatibility. To overcome this challenge, many studies have focused on functionalizing graphene to improve its compatibility with polymer matrices. For example, Gu et al. investigated the functionalization of graphene with a titanate coupling agent and found that it significantly improved the thermal conductivity of the resulting composites. Specifically, the thermal conductivity of the PPS matrix was found to be 19 times higher after the addition of functionalized graphene than that of the pure PPS matrix [21]. Xing et al. developed a method to modify graphene by treating it with 1,3-dihexadecyl-3H-benzimidazolium bromide, resulting in functionalized graphene which was used as a filler in PPS. The results of their study showed that the addition of functionalized graphene can serve as a heterogeneous nucleating agent, leading to an improvement in the crystallinity and fracture strength of the resulting composite. However, it was also observed that the elongation at break of the composite material was significantly reduced [22].

However, most of the research on graphene blended with PPS is focused on the enhancement of the electrical, thermal, or mechanical properties of PPS, and there are very few studies on barrier and antibacterial performance. Additionally, there is no study where nano zinc oxide-decorated graphene (G-ZnO) is used as a filler for PPS to prepare nanocomposites. In this work, PPS/G-ZnO nanocomposites with different proportions were prepared by melt blending, and then the mechanical properties, crystallinity, thermal properties, morphology, and antibacterial activity of the nanocomposites were investigated and explored. The purpose of this work is to obtain a new antibacterial nanocomposite with excellent comprehensive performance through many analyses and the comparison of numerous tests. It is expected that this novel composite material can be applied in the fields of engineering plastics, packaging or medical materials.

## 2. Experimental

### 2.1. Materials

Industrial grade CB602 PPS was procured from Sichuan Zhongke Xingye Materials Co. Ltd., located in Sichuan, China. Nano zinc oxide (ZnO) was acquired from Sichuan Zhixiangyi Technology Co., Ltd. (Chengdu, China). Graphene was provided by Lin Go Industrial Co., Ltd. (Taoyuan, Taiwan). Beef extract, agar, and peptone were purchased from Beijing Oboxing Biotechnology Co., Ltd (Beijing, China).

### 2.2. Preparation of Graphene-ZnO

Nano zinc oxide (ZnO) was acquired from Sichuan Zhixiangyi Technology Co., Ltd. (Sichuan, China). ZnO was coated onto the graphene surface using the wetting dispersion method, with a graphene to ZnO ratio of 4:6. Graphene was dispersed in an ethanol solution with the aid of an ultrasonic bath for 90 min. Next, ZnO was added to the graphene-ethanol mixture, which was then ultrasonicated for 2 h to ensure proper dispersion of ZnO ZnP. The mixture was then filtered to separate the ethanol. To remove any residual ethanol, the mixture was placed in an oven at 40 °C for 4 h and further dried for an additional 1.5 h at 106 °C.

### 2.3. Preparation of PPS/G-ZnO Nanocomposites

**Figure 1:**
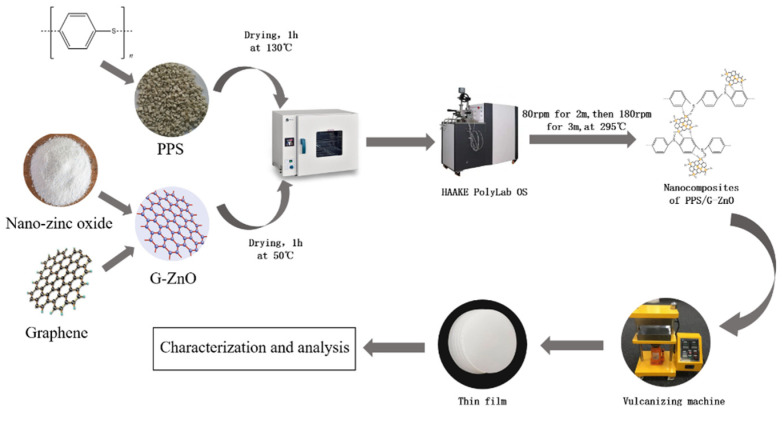
Diagram for processing PPS and PPS/G-ZnO nanocomposites.

Figure 1 depicts the process of preparing PPS/G-ZnO nanocomposites. To begin with, PPS and G-ZnO were individually oven-dried at 130 °C and 50 °C for 4 h, respectively. The nanocomposites were then produced by mixing varying proportions of G-ZnO and PPS in an internal mixer (HAAKE PolyLab OS) under specific conditions, including low-speed mixing at 80 rpm for 2 min, followed by high-speed mixing at 180 rpm for 3 min. The cavity temperature was maintained at 295 °C, and the G-ZnO content ranged from 0.1% to 0.5%. The mixing process involved first adding the PPS matrix and monitoring the torque–time curve. When the curve exhibited slight fluctuations and approached stability, G-ZnO nanomaterials were rapidly added to minimize internal mixing rotor loss and ensure homogeneity. Once the internal mixing was completed, a file was used to remove the PPS/G-ZnO melt from the internal mixing chamber and rotor, and the instrument was cleaned. The composition and quantity of each component used in the preparation of the samples are summarized in Table 1.

### 2.4. Mechanical Performance Test

To evaluate the mechanical properties of PPS and PPS/G-ZnO nanocomposites, dumbbell-shaped samples were prepared by hot pressing and cutting with a knife. These samples were then tested using a tensile testing machine (BS10KNW electronic universal testing machine, Xiamen Forbes Tensile Equipment Co., Ltd., Xiamen, China) according to the Chinese standard GB/T1040-92. The tensile rate was set at 20 mm/min. Data on the mechanical properties of a minimum of 5 samples were obtained, and the average result was calculated. The evaluation of the mechanical properties involved obtaining data on the tensile strength and elongation at break for each sample.

### 2.5. FT-IR Test

An FTIR spectroscopy instrument (Thermo, New York, NY, USA) was utilized to analyze the samples; the test range was 1700–600 cm^−1^; a resolution of 4 cm^−1^ was set. To prepare the PPS and PPS/G-ZnO nanocomposites for Fourier-transform infrared spectroscopy analysis, they were first cut into 11 mm × 11 mm square films, dried in a vacuum drying box at 130 °C for 8 h, and stored in a self-sealing bag to prevent water absorption. Fourier-transform infrared spectroscopy was used in transmission mode to obtain the spectra of the specimens over a range from 4000 to 500 cm^−1^, with 32 scans averaged and a resolution of 2 cm^−1^. This method was employed to identify the characteristic peaks in the IR spectra of the samples.

### 2.6. Morphological Characterization

To observe the cross-section of fractured PPS and PPS/G-ZnO samples, scanning electron microscopy (SEM) was employed using a VEGA 3 SBU instrument from Tescan, Bruker, Czech Republic. Samples were cut to a size that could be properly positioned on a sample holder for effective observation under the electron microscope. Both heat-treated and untreated samples containing various proportions of PPS and G-ZNO were tested. To obtain clear morphological images of the samples at various magnifications, the brightness and focal length of the scanning electron microscope were adjusted accordingly. The micrograph of each sample at 1 kX was selected for analysis.

### 2.7. DSC Analysis

To determine the crystallization temperature and crystallinity of the composite materials, a thermal analysis instrument (DSC200 F3, NETZSCH, Bavaria, Germany) was used. Prior to testing, each sample was weighed at 8 mg. During the test, the temperature was raised from room temperature to 325 °C at a rate of 10 °C/min. The heating process was stopped, and the temperature was held at 325 °C for 10 min before allowing the temperature to decrease naturally. Subsequently, the temperature was increased to 325 °C at a rate of 10 °C/min, and the sample thermal analysis data were obtained.

The parameters of the test sample, such as T crystallization, T melting, and T decomposition, were obtained after the test sample underwent melting, crystallization, oxidation, and decomposition processes. The crystallinity (Xc) was calculated using the following formula:

$$X_{c}=\frac{\Delta H_{m}}{\left(1-\alpha \right)\Delta H_{m}^{0}}\times 100\%$$

where α = filler content, %;

∆*H_m_* = measured enthalpy of fusion, J/g;

∆*H_m_*^0^ = theoretical enthalpy of PBAT at 100% crystallization, 80 J/g.

### 2.8. Thermogravimetric Analysis

To determine the thermal properties of the PPS and PPS/ZnO-0.5 nanocomposites, thermogravimetric analysis (TGA) was performed using an HTG-1 instrument from Beijing Hengjiu, Beijing, China. A 10 mg untreated PPS sample was weighed and placed in a crucible, which was then inserted into the TGA. The sample was heated under a nitrogen atmosphere at a rate of 10 °C/min up to 200 °C, where it was held for 10 min before being further heated to 800 °C at the same rate. The thermogravimetric losses and temperature changes were recorded, and orthogonal curves were plotted to extract the data. A similar procedure was followed for heat-treated PPS and untreated and heat-treated PPS/ZnO-0.5 nanocomposites. A total of four sample groups were measured.

### 2.9. X-ray Diffraction Characterization

Prior to analysis, test samples were dried in an 80 °C oven for 4 h. The composite films were then examined using an X-ray diffractometer (D2-PHASER, Bruker, Billerica, Germany), which allowed for the identification of the corresponding peak values for the composite material. The diffractometer employed Ni-filtered Cu-Kα radiation, and the X-ray beam was directed at the material surface within a range of 2θ = 5–60°. The scan time interval was set at 0.2 s, and the step size was set at 0.02°. The diffraction data for both PPS and PPS/G-ZnO nanocomposites were collected, and the corresponding peaks were identified.

### 2.10. Water Vapor Permeability Coefficients

The water vapor permeability coefficients (WVPC) of the nanocomposite films were evaluated using a Languang W3/060 water vapor transmission rate test system, which employed a vapor-permeable cup weighing method. Prior to testing, the sample films were passed through a vulcanizer, and five samples were prepared for each nanocomposite. The WVPC test was conducted using the following parameters: test chamber humidity of 90%, testing temperature of 37.8 °C, and a test interval of 5 h per time (5 times for each sample). The WVPC values for the samples were then recorded.

### 2.11. Water Contact Angle

To measure the contact angle of PPS and PPS/G-ZNO, several samples were prepared, and a flat surface was selected for the contact angle measuring instrument (JC2000D, Zhongchen Digital Technology Equipment, Shanghai, China). Five nanocomposite films were tested at a measurement temperature of 25 °C. To eliminate the influence of gravity on the contact angle, approximately 2 μL of water droplets from a micro syringe was used. The contact angle was measured when the water droplets made contact with the sample surface. For each nanocomposite film, contact angle measurements were conducted 4 to 5 times at different points, and the average value of the measured contact angle was calculated.

### 2.12. Antibacterial Test

Frozen *Escherichia coli* was activated, then the bacterial solution from the culture solution was prepared with a certain concentration of *Escherichia coli*, and the culture was incubated at 37 °C for 24 h. PPS containing different concentrations of G-ZnO were prepared on diskettes with similar shapes and sizes. The temperature was set at 37 °C, and the shaking speed was 100 rpm. After the cultivation of the culture for 1 d, the same amount of bacterial liquid was taken and diluted following a 10-fold dilution process. The number of dilutions with the highest dilution concentration that could separate a single colony was selected as the reference sample, and the number of a single colony under the same dilution ratio was obtained.

## 3. Results and Discussion

### 3.1. Mechanical Properties

**Figure 2:**
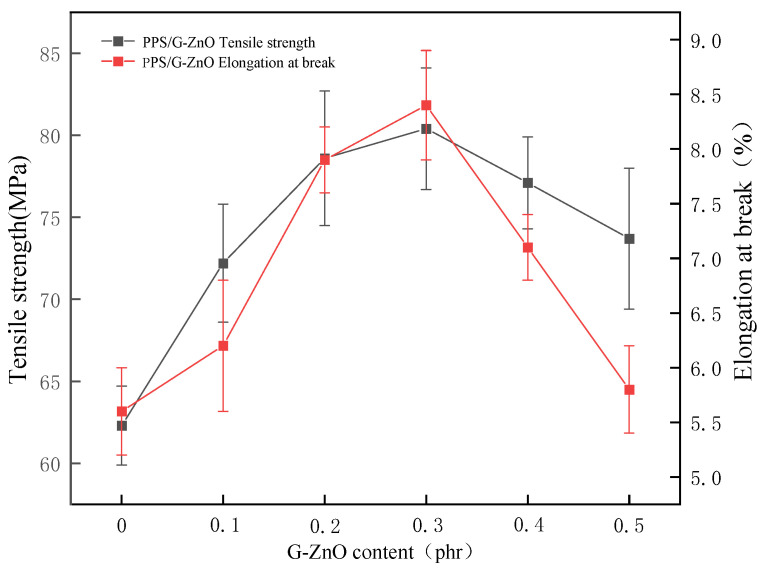
The tensile properties and elongation at break of PPS and PPS/G-ZnO nanocomposites.

Figure 2 shows the tensile properties of PPS and PPS/G-ZnO nanocomposites. Figure 2 indicates that the tensile strength and fracture elongation increase with the increase in nanofiller content when the addition amount of G-ZnO is less than or equal to 0.3%.

G-ZnO can enhance the tensile performance of PPS. This is because graphene has excellent mechanical properties that can improve the strength and stiffness of PPS [23]. In addition, the presence of ZnO can coordinate with PPS and improve the compatibility between graphene and PPS, thereby enhancing the interfacial adhesion between them, preventing material delamination or cracking during tensile testing [24]. When the nanofiller content is too high (0.4 and 0.5%), it may have a negative impact on the material properties due to exceeding the optimal nanofiller content [25]. Excessive nanofiller content can increase the collision and contact between the fillers, thus increasing the cohesion and agglomeration of the fillers [26]. This can lead to poor dispersion of the fillers, which weakens adhesion between the fillers and the matrix, and thus reduces the tensile performance of the material [27].

### 3.2. FTIR

**Figure 3:**
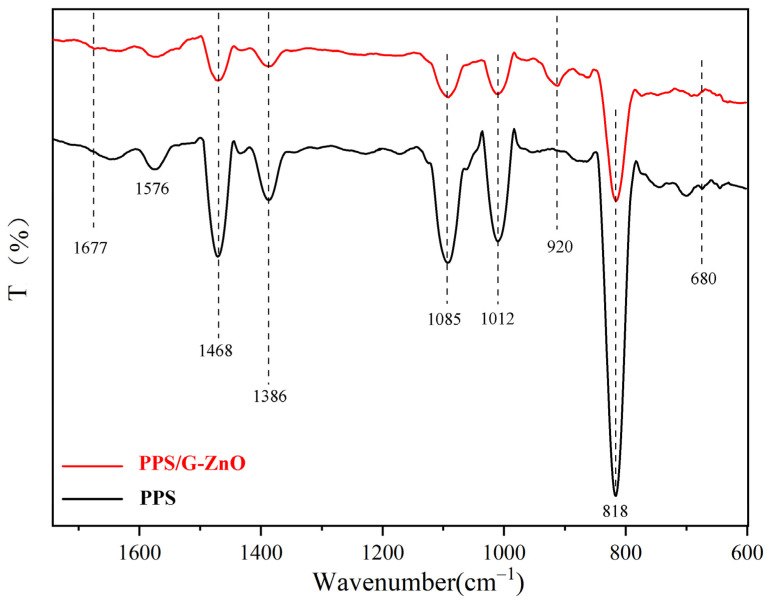
FTIR spectra of PPS and PPS/G-ZnO nanocomposites.

Figure 3 shows the infrared spectral curves of PPS and PPS/G-ZnO nanocomposites, where the peaks observed in neat PPS were induced by the aromatic C=C bond [28] and vibrations of C-S [29] and C-H [30]. In comparison, the C-S stretching vibration band of the PPS/G-ZnO nanocomposite appears at a lower frequency, which could be due to partial cross-linking or steric hindrance of the benzene ring. A new band of S=O double bond stretching vibration at 920 cm^−1^ also appears in the nanocomposite material due to -S-O- cross-linking [31,32].

**Figure 4:**
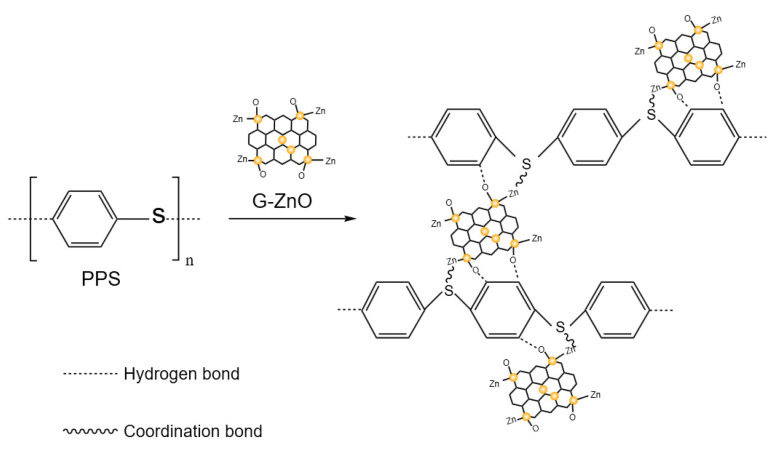
Possible reaction mechanism of PPS and PPS/G-ZnO nanocomposites.

Significantly, upon addition of G-ZnO to PPS, there were noticeable changes in the infrared spectral curves. The peak (818 cm^−1^) of relative intensity observed in PPS/G-ZnO became smaller compared to neat PPS. Additionally, the relative intensity of the peak at the position of 1650 cm^−1^ decreased, whereas the C-H relative intensity at the peak position of 1677 cm^−1^ increased. These changes could be attributed to the interaction between the H from C-H of PPS and ZnO from G-ZnO through hydrogen bonding. Figure 4 presents the possible reaction mechanisms for the formation of the network structure between PPS and G-ZnO involving both hydrogen bonds and coordination bonds.

### 3.3. SEM

**Figure 5:**
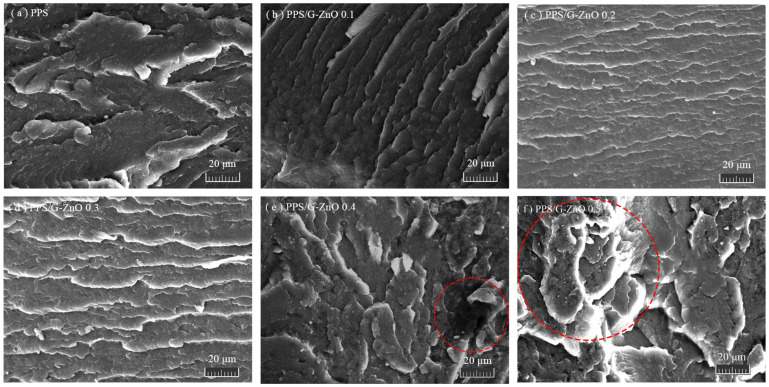
Scanning electron micrographs.

Figure 5 shows the SEM images of the tensile fracture surface of PPS and PPS/G-ZnO nanocomposites. For the morphology of neat PPS revealed in Figure 5a, the fractured section is very rough, which may be because of incomplete crystallization, the proportion of the indeterminate phase is large, and the fracture surface presents large cracks [33]. This morphology is similar to the morphology of a fractured section of neat PPS obtained in a previous work [34,35]. With the addition of 0.1%, 0.2% and 0.3% G-ZnO to the PPS matrix, relatively flat fractured sections appear, as shown in Figure 5b–d, and a layered structure with similar toughness properties is also presented, which may be attributed to the uniform dispersion of nanofiller and a wider distribution (especially when G-ZnO = 0.3%), making G-ZnO and PPS become a staggered 3D network structure. Therefore, when G-ZnO is 0.3%, its structure is more compact, and G-ZnO also achieves the best reinforcement effect, greatly improving the tensile strength and elongation at break of PPS. When the concentration of CG reaches 0.4%, its fracture surface becomes rough, and some protrusions appear (Figure 5d); it may be that G-ZnO is not easily dispersed at 0.4% concentration, resulting in the slight aggregation of nanofillers. When the concentration of G-ZnO further increased to 0.5%, the cross-section morphology of its nanocomposites changed significantly. Bigger protrusions appear on the fracture surface, and an even larger area of deformation can be seen (see the red dashed circle shown in Figure 5e,f). This is the phenomenon of incompatibility of nanocomposites due to a large amount of G-ZnO agglomeration. The above situation corresponds to the test results for tensile properties. This further confirms the changes in tensile properties.

### 3.4. DSC Analysis

**Figure 6:**
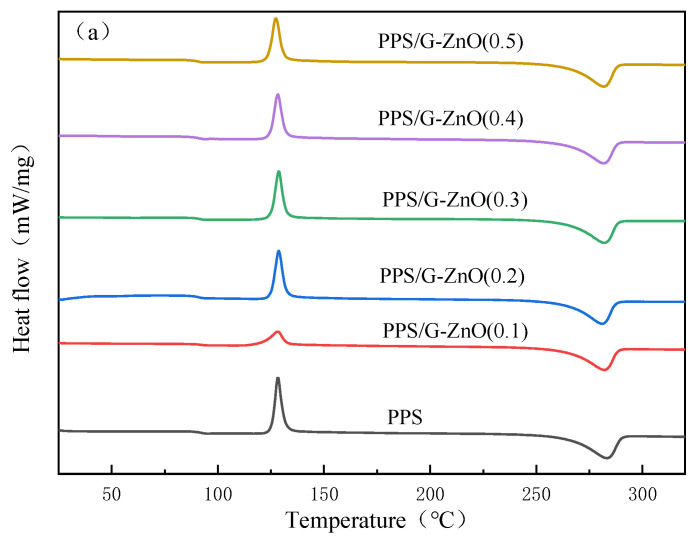

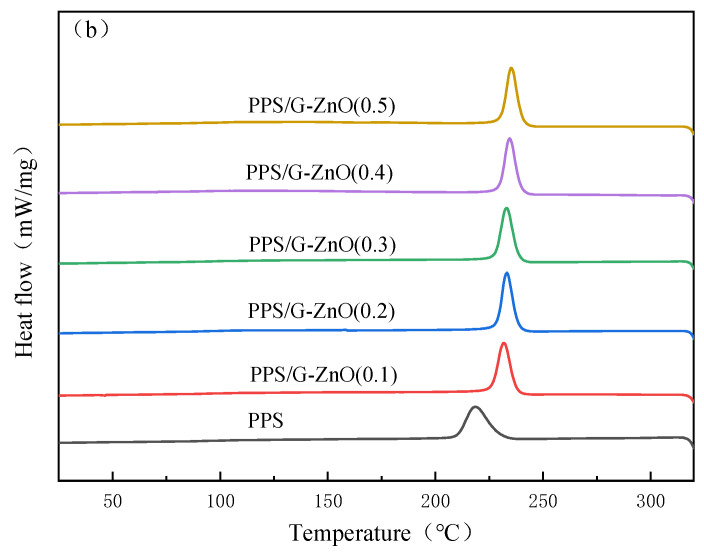
Differential scanning calorimetry for PPS and PPS/G-ZnO nanocomposites. (**a**) Second heating curves; (**b**) Cooling curves.

Figure 6a,b show the DSC (differential scanning calorimetry) second heating and cooling curves of neat poly (phenylene sulfide) (PPS) and PPS/G-ZnO nanocomposites, respectively, and detailed data are listed in Table 2. It can be observed from Figure 6 and Table 3 that the melting temperature (Tm) of PPS decreases with increasing concentration of G-ZnO, and the crystallization temperature (Tc) decreases with increasing concentration of G-ZnO. This may be attributed to the fact that the nanofiller can promote the nucleation of PPS [36,37]. As the concentration of G-ZnO increases, the number of nuclei increases, which increases the crystallization rate but hinders the growth of nuclei, resulting in smaller crystalline sizes [38,39]. As a result, PPS/G-ZnO nanocomposites are more likely to melt at lower temperatures during the heating process.

Table 2 also indicates that the crystallinity exhibits an initial increase and then a decreasing trend, reaching its maximum value at G-ZnO concentrations of 0.2% and 0.3%, respectively. These two values are very close, and the trend is similar to that observed in the mechanical properties. Therefore, the change in crystallinity may be related to the mechanical properties of the nanocomposites.

### 3.5. XRD

**Figure 7:**
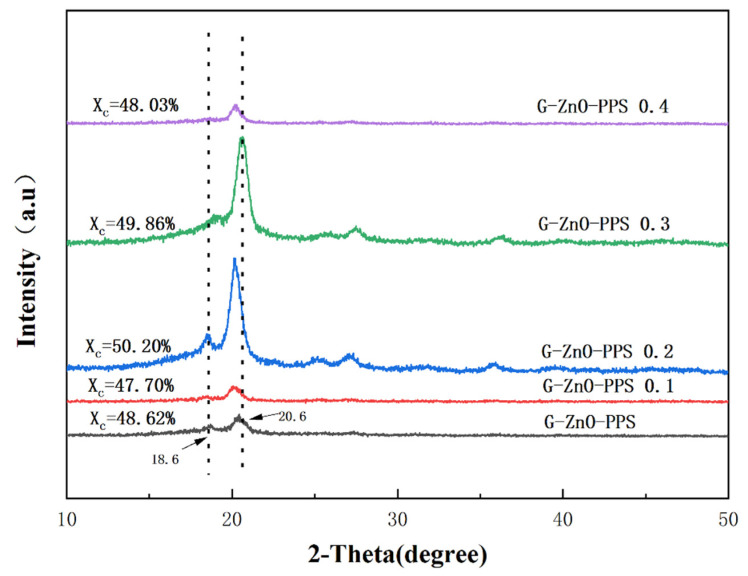
X-ray diffraction of PPS and PPS/G-ZnO nanocomposites.

Figure 7 shows the XRD (X-ray diffraction) spectra of neat PPS and PPS/G-ZnO nanocomposites. The characteristic diffraction peaks of PPS and its nanocomposites are similar, indicating a similar crystal structure. The strong diffraction peaks at 2θ = 18.6° and 20.6° correspond to the (110) and (002) crystal planes, respectively [40,41,42]. The nanocomposite crystallinity reached its maximum when the G-ZnO concentration was 0.2%, which may be due to the saturation of uniformly dispersed nanomaterials in the PPS polymer matrix [43,44]. The intensity of the peak and the value of crystallinity were similar when the G-ZnO concentration was increased to 0.3%, indicating good dispersion. It is worth noting that the XRD crystallinity trend is almost identical to the DSC crystallinity trend, and similarly, the values of G-ZnO concentration in the nanocomposites at 0.2% and 0.3% are also very close, further demonstrating the reliability of both crystallinity values. However, at G-ZnO concentrations of 0.4%, the intensity of the peak decreased significantly due to agglomeration within the crystal gaps, which caused unbalanced crystal growth and a decrease in crystallinity [45]. The characteristic diffraction peaks of PPS/G-ZnO nanocomposites shifted to the left, except for the two peaks that shifted to the right when the G-ZnO concentration was 0.3%. This shift to the left may be attributed to an increase in distances in the interplanar spacing and lattice parameter [46]. The excellent dispersion of the nanofiller at 0.3% concentration resulted in the widest and most extensive distribution in PPS, leading to a decrease in crystal plane distance and achieving the highest tensile performance.

### 3.6. TGA

**Figure 8:**
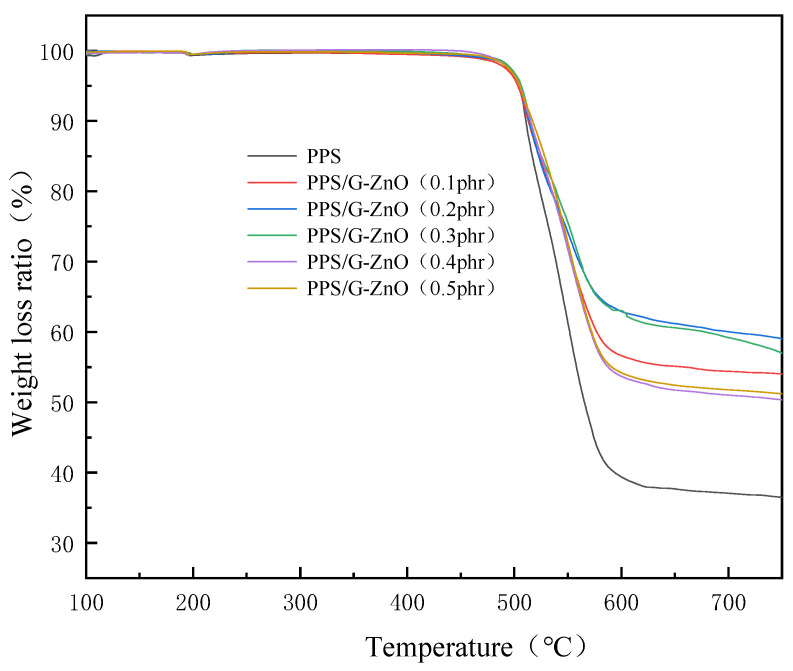
Thermogravimetric curves for PPS and PPS/G-ZnO nanocomposites.

Figure 8 and Table 3 present the TGA (thermogravimetric analysis) curves and detailed numerical data, respectively. From Table 3, it can be observed that the addition of G-ZnO to PPS showed a slight increase in temperature at 10% weight loss, and the weight-loss difference of all nanocomposite samples was not significant. This also indicates that the presence of nanofillers has a positive impact on the initial thermal degradation temperature of PPS. Table 3 shows that the maximum degradation temperature (T_max_) initially increases and then decreases with increasing G-ZnO concentration, reaching their maximum values at 0.2% and 0.3% G-ZnO concentration. This can be attributed to two factors: (1) the surface of G-ZnO can adsorb polymer chains and form a barrier, slowing down the propagation rate of the thermal decomposition reaction [47]; and (2) G-ZnO has high thermal conductivity, which promotes more uniform heat transfer in the material, thus improving the thermal stability of PPS [48]. When the G-ZnO concentration exceeds 0.3%, the thermal decomposition temperature shows a decreasing trend. This may be due to poor dispersion of G-ZnO in the PPS matrix, resulting in poor compatibility between the nanofiller and the polymer, which could increase the interfacial stress and decrease the thermal stability of PPS [49]. The TGA analysis results show similar trends to those observed in the mechanical properties and DSC crystallinity, suggesting that the crystallinity of PPS/G-ZnO nanocomposites may also affect their mechanical properties and thermal stability, in addition to the dispersion of G-ZnO within the polymer matrix.

### 3.7. Water Vapor Permeability Coefficients

**Figure 9:**
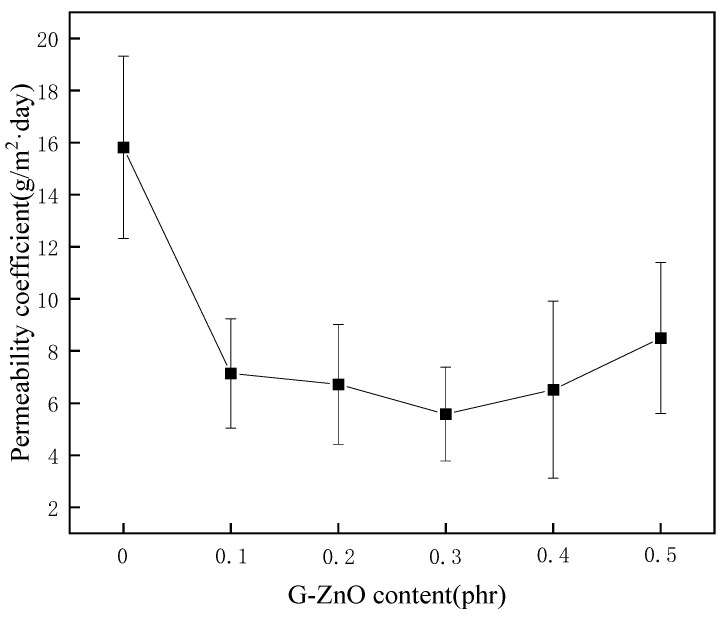
The permeability coefficient of PPS and PPS/G-ZnO nanocomposites.

**Figure 10:**
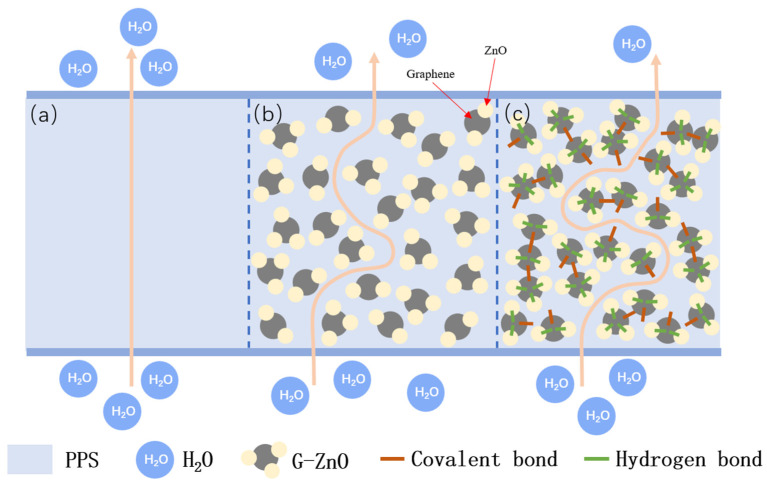
Schematic diagram of H2O molecules diffusing through PPS and PPS/G-ZnO nanocomposites: (**a**) diffusion path through PPS; (**b**) diffusion path around PPS/G-ZnO nanocomposite; (**c**) the narrower diffusion path around PPS/G-ZnO nanocomposites.

Figure 9 exhibits the average water vapor permeability coefficients (WVPC) of PPS and PPS/G-ZnO nanocomposites at G-ZnO changed concentration. It can be observed from Figure 8 that the WVPC exhibits a valley-like trend. When the concentration of G-ZnO is from 0.1 to 0.3%, the WVPC of nanocomposites are lower than that of neat PPS, which means the barrier properties for those of the nanocomposites are better. When the concentration reaches 0.3%, the WVPC is lowest at 5.58 g/m^2^·day. This may be attributed to the existence of G-ZnO, which makes the path of H_2_O through the nanocomposites more complicated than in neat PPS and increases its barrier performance [50,51]. For pure PPS, H_2_O molecules would be advanced along a vertical path (see Figure 10a). Whereas in PPS/G-ZnO nanocomposite, the H_2_O molecules may not pass through the nanofillers and change direction around the G-ZnO (see Figure 10b) [52]. Furthermore, the Zn ion of ZnO from G-ZnO produces coordination bonds with O atoms of PPS polymer matrix, thus making the H_2_O transmission path narrower (see Figure 9c) [53]. However, when the concentration of G-ZnO was 0.4%, the WVPC become larger. This is because G-ZnO causes defects in the nanocomposites, which allow H_2_O molecules to pass through the PPS/G-ZnO nanocomposite more easily.

### 3.8. Contact Angle

**Figure 11:**
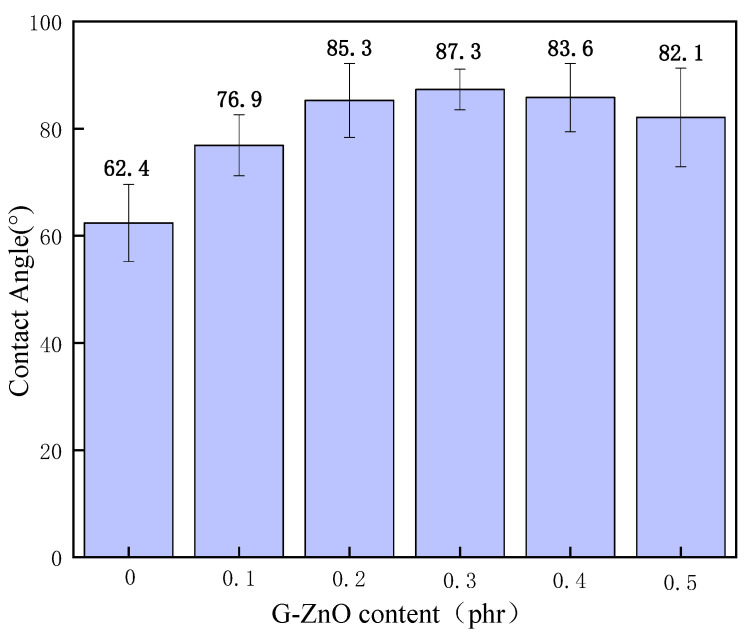
Contact angle of PPS and PPS/G-ZnO composites.

Figure 11 shows the contact angle (CA) for PPS and PPS/G-ZnO nanocomposites. The figure demonstrates the CA tests, in which water droplets were placed onto the surface of PPS and PPS/CG nanocomposite films for 20 s. With an increase in G-ZnO concentration, the CA values of the PPS increase significantly when G-ZnO is added to PPS ≤ 0.3% and reaches the maximum value at 0.3%. This may be because G-ZnO nanofillers are uniformly and widely dispersed in the PPS polymer, so the PPS/G-ZnO is the most compact in structure and displays greater hydrophobicity when G-ZnO was incorporated with PPS at 0.3% [54]. When the addition of G-ZnO was greater than 0.3%, the CA value decreased with the increase in addition.

This is in agreement with the results from WPVC and TGA testing. When G-ZnO concentrations are more than 0.3%, an uneven dispersion appears in the polymer matrix. The structures of the PPS/ G-ZnO nanocomposites have more flaws, and the H_2_O molecules can more easily enter the nanocomposite samples. The results from CA show that the hydrophobicity and structure of compactness for PPS can be enhanced by the addition of the optimum content of G-ZnO.

### 3.9. Antibacterial Test

**Figure 12:**
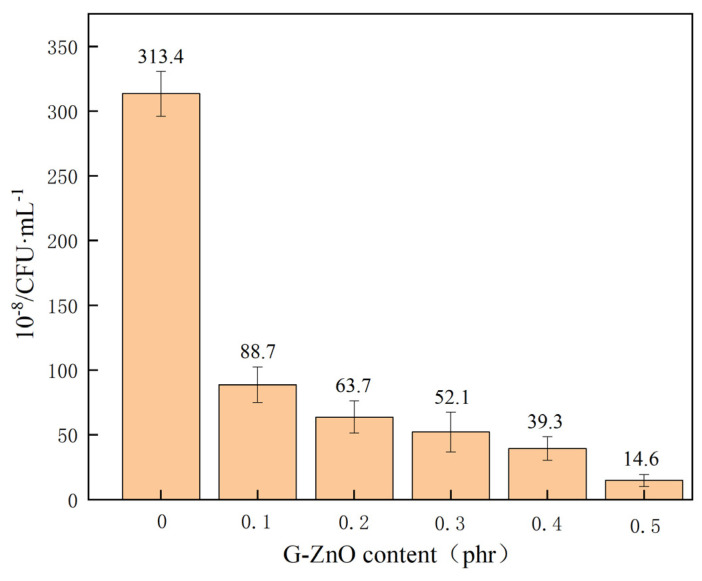
Colony forming unit of PPS and PPS/G-ZnO composites.

Figure 12 shows an examination of the antibacterial properties for neat PPS, and PPS/G-ZnO nanocomposites, against *E. coli*. After G-ZnO was incorporated into PPS, the PPS/G-ZnO nanocomposites showed a smaller number of viable bacteria. As the concentration of G-ZnO increased, the number of bacteria decreased. This can be attributed to the cytotoxicity of graphene to bacteria [55] and the antibacterial properties of ZnO [56,57] due to its highly active surface. Graphene and ZnO can interact with bacterial cell membranes, leading to the destruction of bacterial structures and metabolic processes, ultimately resulting in bacterial death [58]. The small size and high specific surface area of nano zinc oxide contribute to its efficacy. Furthermore, the release of zinc oxide ions from nano zinc oxide can also kill bacteria by oxidizing and dissociating proteins, nucleic acids, and other biological molecules, thereby having a bactericidal effect. It is worth noting that the use of G-ZnO as an antibacterial agent for PPS has been extensively explored in various fields, including healthcare, food packaging, textiles, and building materials, among others.

## 4. Conclusions

PPS nanocomposites were reinforced with G-ZnO as multipurpose additives. FTIR analysis showed that G-ZnO interacts with PPS through coordination and hydrogen bonds. Studies on tensile properties revealed significant improvements in the tensile strength and elongation at break of the nanocomposite after the addition of G-ZnO. XRD analysis indicated that G-ZnO nanomaterials cause changes in some crystal forms and greatly improve the crystallinity of PPS. The optimal G-ZnO concentration was found to be 0.3%, and when the nanofiller is uniformly dispersed, the nanocomposite exhibits maximum tensile strength, elongation at break, thermal stability, barrier property, and hydrophobicity. Incorporating G-ZnO into PPS at a ratio of 0.3% resulted in significant improvements in the tensile strength, elongation at break, and WVPC. Specifically, compared to pure PPS, the tensile strength increased from 62.3 to 80.4 MPa, and the elongation at break increased from 5.6 to 8.4 MPa. The antibacterial nanocomposites prepared by the addition of G-ZnO to PPS exhibit excellent comprehensive performance, indicating their potential in engineering, medical materials, and high-temperature resistant materials.

## Figures and Tables

**Table 1:** Proportions of PPS and G-ZnO nanocomposites.

Sample	PPS (%)	G-ZnO (%)
PPS	100	0
PPS/G-ZnO_0.1	99.9	0.1
PPS/G-ZnO_0.2	99.8	0.2
PPS/G-ZnO_0.3	99.7	0.3
PPS/G-ZnO_0.4	99.6	0.4
PPS/G-ZnO_0.5	99.5	0.5

**Table 2:** DSC parameters for PPS and various PPS/G-ZnO nanocomposites.

Sample	Second Heating			Cooling
	T_m_ (°C)	ΔH_m_ (J/g)	Xc (%)	T_c_ (°C)
PPS	283.1	−38.9	48.62	218.6
PPS/G-ZnO_0.1	282.0	−38.12	47.70	231.8
PPS/G-ZnO_0.2	280.8	−40.08	50.20	233.2
PPS/G-ZnO_0.3	282.0	−39.77	49.86	233.1
PPS/G-ZnO_0.4	281.5	−38.27	48.03	234.5
PPS/G-ZnO_0.5	281.7	−39.0	48.99	235.2

**Table 3:** TGA data for PPS and various PPS/MWCNTs-ZnO nanocomposites.

Sample	T_10%_ (°C)	T_max_ (°C)	M_e_ (%)
PPS	507.7	511.2	35.7
PPS/G-ZnO_0.1	508.0	510.8	54.1
PPS/G-ZnO_0.2	509.2	514.2	57.0
PPS/G-ZnO_0.3	509.5	514.2	54.0
PPS/G-ZnO_0.4	509.6	510.1	49.4
PPS/G-ZnO_0.5	510.5	507.5	50.4
